# Spontaneous slow oscillation—slow spindle features predict induced overnight memory retention

**DOI:** 10.1093/sleep/zsab127

**Published:** 2021-05-18

**Authors:** Fereshteh Dehnavi, Ping Chai Koo-Poeggel, Maryam Ghorbani, Lisa Marshall

**Affiliations:** 1 Department of Electrical Engineering, Ferdowsi University of Mashhad, Mashhad, Iran; 2 Institute of Experimental and Clinical Pharmacology and Toxicology, University of Lübeck, Lübeck, Germany; 3 Center of Brain, Behavior and Metabolism, University of Lübeck, Lübeck, Germany; 4 Rayan Center for Neuroscience and Behavior, Ferdowsi University of Mashhad, Mashhad, Iran

**Keywords:** learning and memory, slow wave sleep, sleep spindles, slow oscillation slope, phase amplitude coupling

## Abstract

**Study Objectives:**

Synchronization of neural activity within local networks and between brain regions is a major contributor to rhythmic field potentials such as the EEG. On the other hand, dynamic changes in microstructure and activity are reflected in the EEG, for instance slow oscillation (SO) slope can reflect synaptic strength. SO-spindle coupling is a measure for neural communication. It was previously associated with memory consolidation, but also shown to reveal strong interindividual differences. In studies, weak electric current stimulation has modulated brain rhythms and memory retention. Here, we investigate whether SO-spindle coupling and SO slope during baseline sleep are associated with (predictive of) stimulation efficacy on retention performance.

**Methods:**

Twenty-five healthy subjects participated in three experimental sessions. Sleep-associated memory consolidation was measured in two sessions, in one anodal transcranial direct current stimulation oscillating at subjects individual SO frequency (so-tDCS) was applied during nocturnal sleep. The third session was without a learning task (baseline sleep). The dependence on SO-spindle coupling and SO-slope during baseline sleep of so-tDCS efficacy on retention performance were investigated.

**Results:**

Stimulation efficacy on overnight retention of declarative memories was associated with nesting of slow spindles to SO trough in deep nonrapid eye movement baseline sleep. Steepness and direction of SO slope in baseline sleep were features indicative for stimulation efficacy.

**Conclusions:**

Findings underscore a functional relevance of activity during the SO up-to-down state transition for memory consolidation and provide support for distinct consolidation mechanisms for types of declarative memories.

**Statement of Significance**

Memory consolidation requires local and interregional network activity. The temporal coupling of sleep spindles to the slow oscillation, and slow oscillation slopes are taken to reflect underlying network interactions, and neural synchronization processes, respectively. Here, using correlation analyses, we show slow oscillation-spindle coupling measures during nonlearning baseline slow wave sleep are indicative for the efficacy of applied weak electric current stimulation. Stimulation efficacy on figural and word paired-associate tasks were differentially related to SO slope, further suggesting a functional relevance of baseline rhythmic properties.

## Introduction

The consolidation of hippocampus-dependent memories is assumed to rely on the interplay of hippocampal and thalmo-cortical activity, as deduced from studies decreasing or increasing related neural communication [1–3]. The temporal relationships of hippocampal sharp-wave ripples, thalamo-cortical spindles and neocortical slow waves or slow oscillations present an indication of successive of regional neuronal interactions. Yet, despite concrete findings on relationships between neuronal unit activity and oscillatory field rhythms, the neural processes underlying memory consolidation remain mostly obscure. The complexity of potential mechanisms is underscored by recent findings revealing bidirectional (cortico-hippocampal) interactions during sleep [4–6].

Many faster neural rhythms or events couple to a predominant (or “preferred”) phase of slower sleep oscillations. In general, neural coupling is found between spike firing and synchronized population activity, or between faster frequency local activity and larger-scale slower activity, thus coupled EEG oscillations reflect mechanisms to transfer neural information across different organizational levels [7–9]. SO-spindle coupling may prove a more sensitive marker than EEG rhythms per se for the susceptibility to so-tDCS neuromodulation. During sleep coupling is observed between the slow oscillation: sleep spindles, hippocampal and cortical ripples, and theta bursts [6, 10] Human scalp EEG recordings typically permit only the measurement of SO-spindle coupling, although ripples in scalp recordings of potential physiological relevance have been reported [11, 12].

Sleep spindles present the first scalp electrophysiological event of nonrapid eye movement (NREM) sleep for which an association to sleep-associated memory consolidation was found [13–15]. In human sleep, spindles within two frequency ranges are typically discerned, slow (9–12 Hz) and fast (12–15 Hz) [16, 17]. For fast spindles, in addition to modifications in features such as density, amplitude and length, which are dependent upon by presleep learning, correlations with trait-like cognitive metrics exist [18–20]. Evidence on the grouping of fast centro-parietal scalp EEG spindles by the SO is most ubiquitous. These thalamocortical fast spindles preferentially couple to the (depolarizing) up-state of the SOs, or down-to-up state transition [21]. Not only the sources and emergence of slow spindles, but also their phenomenological descriptions are less consistent. EEG slow spindles are most pronounced during deep NREM sleep, reveal a more anterior topographical distribution than fast spindles, differ in their phase relation to the SO, occurring mainly during the cortical up to down state transition or down state [22–24], and diverge in their pharmacological responsivity [25]. While growing evidence suggests the importance of SO-fast spindle coupling in the processing of declarative memory [26, 27], the functional relevance of SO-slow spindle coupling is unclear [28, 29].

Relationships between processes of memory consolidation and sleep stages, single brain rhythms or activity within one brain region, are for technical reasons, more commonly investigated than relationships with the temporal coupling of oscillations [30–32]. SO slopes have received less interest regarding memory consolidation. Yet, experimental, and modeling data suggest a linkage between SO slope and synaptic strength or processes of memory consolidation [33–36]. The SO consists of a longer duration up and shorter down state within the range of several hundred milliseconds. Steepness of EEG and local field potential SO slopes correspond to the more synchronized onset of silent and active states and some inferences on cellular activity at the transitions between up and down states can be drawn [36, 37]. SO slopes are reported to change over the course of nocturnal sleep with sleep stage, and reflect in part a homeostatic feature, as sleep pressure and slope showed a positive relationship [38–40]. In one study investigating the interrelationship between SO slope and memory consolidation, Rihm et al. reported that changes in SO slope within the first 10 s of odor presentation for an odor delivered during learning prior to sleep in NREM sleep compared to a previous 10 s nonodor period, predicted memory retrieval after sleep. These increased changes in slope were accompanied by increased fast spindle EEG power [41]. Due to EEG SO slopes and their changes presenting potential measures for the dynamics of cortical network activity, interactions with processes of memory consolidation are potentially very interesting. Moreover, as mentioned above, the sleep spindle types occur during different SO phases, i.e. are coupled to opposite SO slopes. Interestingly, the coupling of sleep spindles to SOs are characterized by strong interindividual variability while intraindividual coupling constellations are most consistent [42].

In several studies, associations between specific baseline EEG measures and cognitive performance or cortical processes have been disclosed [43–46]. In particular, the SOs and slow wave activity of NREM sleep are suggested to forecast cortical processes, mostly related to cognitive decline [43, 47–49] and present a target to affect memory consolidation.

The application of weak electric currents presents one attractive exogenous stimulation procedure found to modulate ongoing brain rhythms and memory consolidation. During sleep anodal slow oscillatory transcranial direct current stimulation (so-tDCS; oscillating at ~0.75 Hz) targets the ongoing SO and associated thalamo-cortical network activity [50, 51], enhancing SO, spindle activity and improving declarative memory consolidation [52–54]. Yet, many results with noninvasive brain stimulation results revealed poor reproducibility [55–58], possibly attributed to pronounced variations in individual network susceptibility and/or electric field distribution [59–62]. In a previous study of ours, differential effects on sleep associated memory retention of weak exogenous electric stimulation (so-tDCS) correlated with subjects’ memory quotient, an index that indicates one´s memory capacity [60]. Here, we implement nonlinear phase dependent correlation measures to examine whether features of SO-spindle coupling and SO slope during baseline sleep (a nonlearning control condition) are associated with (predictive of) stimulation efficacy on retention performance of three declarative tasks. Data in this study were previously used in Koo and colleagues [60].

## Methods

### Participants

Analyzed data were obtained from a study reported elsewhere [60]. In brief, subjects (*n* = 25, female: 15, ranging from 19 to 26 years, mean age: 22.4 ± 2.12 years) were required to be nonsmokers, right-handed, have no metallic implants, nor a history of psychopathological disorders, or any cognitive impairment; be free of medication, and to have a regular sleep rhythm. All participants signed the consent form prior to participation. The study was approved by the local ethics committee of the University of Lübeck, Germany.

### Experimental design and procedure

Subjects underwent an adaptation night in the lab, and subsequently participated in three experimental sessions, separated by at least seven days. In two of the three experimental conditions, a battery of memory tasks was given prior to nocturnal sleep. In one of these nights anodal so-tDCS was applied, the other was a Sham-stimulation session. The third condition consisted of a nonlearning baseline session, positioned between stimulation and sham sessions. This nonlearning, nonstimulation night is taken to represent undisturbed sleep. We assume that parameters assessed during this baseline session reflect a trait or at least a rather stable marker for interindividual differences. We aimed to minimize carryover effects between the memory tasks by prolongation of this intersession interval. Stimulation and sham nights were unblinded to experimenters and pseudo-randomly allocated to either the first or the last session, with their order counterbalanced across subjects.

### Memory tasks

Memory consolidation was assessed using three declarative tasks: word paired-associate, WPA; figural paired-associate, FPA; and the 2D-object location, 2DL. During learning in the FPA task, subjects were presented consecutively with 16 ﬁgural pairs (cue-target). Figures were made up of either geometric or nongeometric lines, with each pair presented for 5 s on the computer monitor followed by a 1 s interstimulus interval. Immediate cued recognition followed presentation of the 16 figural pairs. Subjects were asked to choose the correct target ﬁgure out of eight line-drawings upon presentation of the cue. Their response was followed by the correct answer. There were no time constraints. Learning was repeated until a minimum of 10 correct answers (corresponding to 60%) during immediate cued recognition were given. Sequences of stimulus presentations were balanced across subjects for each learning, immediate and delayed recall. The procedure of delayed cued recognition, conducted after the sleep period, was the same as the immediate cue recognition but without feedback. Performance at immediate and delayed cued recall is termed ‘Learning’ and ‘Recall’ performance, correspondingly.

The WPA task consisted of 80 semantically related German word-pairs (cue-target). Subjects were to memorize two separate lists of 40 word-pairs each, whereby the ﬁrst and last three word-pairs of each list served as dummies and were removed, resulting in a total of 68 word-pairs for analysis. A 2 min break was given between presentation of the ﬁrst and second lists. Word-pairs were presented sequentially on a monitor for 4 s each with an interstimulus interval of 1 s. Immediate cued free recall of all 80 words was performed once after learning (‘Learning’ performance). Delayed recall (‘Recall’) performance was assessed after the sleep period. The order of pairs was randomized during learning; sequences for each immediate and delayed recall remained, however, the same across subjects.

For the 2D-object location task (2DL), 15 pairs of picture cards were presented in a 5 × 6 matrix on the monitor. After initial learning of figure location, subjects were required to indicate the position of the target picture on presentation of the cue. A 60% learning criteria was also used for this task, that is, if subjects failed to reach the criterion, a new learning trial with a different order of objects was initiated [63].

Furthermore, two procedural memory tasks (finger sequence tapping, FST; mirror tracing, MT) were conducted as controls. For the FST subjects were to type on a keyboard as quickly and accurately as possible a sequence of five elements (with numbers from 1 to 4, e.g. 4-1-3-2-4) presented on the monitor. In the MT task subjects were required to trace as fast and accurately as possible a line-drawn meaningless figure. Only mirror images of their hand movements and the figure were visible. Details of these tasks were reported previously [60]. Here, we focus on the modulation of SO-spindle coupling by so-tDCS of the declarative memory tasks since for hippocampus-dependent declarative tasks substantial evidence points toward the relevance of such coupling for memory consolidation [27, 64, 65].

### Sleep monitoring and EEG data acquisition

Raw data were acquired with a DC amplifier, a sampling rate of 500 Hz, with a low-pass filter set at 200 Hz; a gain of 10 dB, an amplitude resolution of 32-bit float values and accuracy of 29.80 nV/LSB (SynAmps RT, Compumedics Neuroscan, Charlotte, USA [60]). For analyses, data were high-pass (0.16 Hz) and low-pass (33 Hz) filtered, off-line re-referenced from the nose to linked mastoids and subsequently downsampled to 100 Hz. All analyses were conducted after down-sampling.

### SO-tDCS

Anodal oscillatory stimulation was applied bilaterally at frontolateral locations (F3, F4; of the international 10:20 system; return electrodes at the corresponding ipsilateral mastoid) and induced by a battery driven customized constant current stimulator with two synchronized circuits. The current of trapezoid shape with equally long plateaus, rising and falling slopes, oscillated between 0 and 300 μA. Impedance was <1 kΩ. Individual slow oscillation stimulation frequencies were obtained from the first cycle of NREM sleep in the adaptation night (0.84 ± 0.02 Hz). Stimulation was applied in five blocks of 5-min followed by at least 1-min stimulation free epochs (or longer if movements were detected and/or subjects transferred into stage N1 sleep or wake; range 59–311 s). The first stimulation block commenced once subjects revealed 4 min of continuous N2 as scored online. Stimulation was only applied when subjects were in sleep stage N2 or deeper. In Sham subjects received only two slow oscillation stimulation cycles (~2.6 s) instead of 5-min of SO stimulation. Stimulation electrodes (8 mm sintered Ag/AgCl) were plugged into a headbox placed next to the subject which was connected to the stimulator located in the observation room. Subjects were asked at the end of each session whether they believed to have received or felt the stimulation [60].

### Behavioral data analysis

Retention was calculated as 100 × (recall performance − learning performance)/learning performance. SO-tDCS efficacy was defined at retention_STIM_ –retention_SHAM_.

### Sleep EEG preprocessing

Movement artifacts were removed through visual inspection. We restricted our analyses to two channels: Fz, for SO-slow spindle coupling and SO slope to, and Cz, for SO-fast spindle coupling to, corresponding to the predominant topographic locations of (global) spindle activity and slow oscillations. Channel substitution was performed for noisy channels based on visual inspection of the individual power spectra of the Fz and Cz channels. For one subject, data of Fz in the Stimulation condition was substituted by data of F7; in another subject Cz was substituted by C3 in the baseline sleep condition.

Sleep stages wake, N1, N2, N3, and REM sleep were determined according to criteria of the American Academy of Sleep Medicine (AASM) manual [66] by two independent scorers. All analyses were conducted with data during N3 from the same 150-min poststimulation time period which began immediately after the termination of stimulation or sham-stimulation as reported in Koo et al. [60]. Similar results for so-tDCS efficacy on retention of the three declarative tasks were found when the entire NREM sleep during the 150-min poststimulation period of baseline was examined. Only EEG data of baseline sleep are used for analyses here.

### Spectral analysis and spindle peak detection

The ranges of the fast and slow spindles were determined separately for each subject from the detected spindle peaks in the power spectrum. First on every artifact-free 5 s block of EEG data, a Hanning window was applied before calculating power spectra of the epoch using fast Fourier transforms; a 50% overlap was used (pwelch MATLAB function). The algorithm for spindle peak detection was similar to that by Cox and colleagues [67]. In brief, peaks within the spindle frequency band were individually determined from power estimates after calculating the temporal derivative of EEG epochs. Since this approach counteracts the 1/f effect in the spectrum and spectral peaks are more easily detected relative to surrounding frequencies [68]. Thereafter, data were smoothed using a moving window of 0.6 Hz, and the power spectrum of each electrode was normalized by dividing each data point of the spectrum by the average power in the 0.2–4 Hz frequency band of the corresponding electrode. Finally, for spindle peak detection, each power spectrum was rescaled between the minimum and maximum values in the frequency ranges below 20 Hz. Spindle peaks were determined during N3 and if required during N2 across Fz for slow spindles, and across Cz for fast spindles. Spectral peaks were detected using Matlab function “findpeaks” with a minimum prominence setting of 0.02 in the frequency range of 9–11.5 Hz for slow spindles and 12.5–15 Hz for fast spindles. In addition, peaks within the frequency range of 5–8 Hz of NREM sleep were detected. For subjects without a prominent discrete slow spindle peak in stage N3 of baseline sleep, the average slow spindle peak frequency value for the other two conditions was used. On absence of any prominent discrete slow spindle peak in N3, the peak frequency in N2 was used. When a clear slow spindle peak was not discernable in any condition or stage, the average slow spindle peak frequency across all other subjects was used. All analyses were conducted with individual spindle peak frequencies. Peak frequencies of fast and slow spindles did not significantly differ between the three conditions. Individual slow and fast spindle frequency ranges were determined as ±1 Hz around the peak frequency. For 12 (21) of the 14 (25) subjects the frequency range of slow (fast) spindles was below (above) 12 Hz. To avoid overlap between the two spindle types for the remaining subjects, the individually determined frequency ranges for slow and fast spindle frequency bands were limited to a maximum of 12 Hz or a minimum of 12 Hz, respectively. A similar procedure was used to detect the theta peak frequency ranges. For subjects without a prominent discrete theta peak in stage N3 of baseline sleep, the theta peak frequency value from Sham during N3 was used. On absence of any peak in stage N3 of Sham the peak frequency in N2 was used. Finally, when a theta peak was not discernible the average value for theta peak frequency across subjects was used.

### Slow oscillation detection

The algorithm for detecting SO was similar to that by Klinzing [24]. In brief, the down-sampled EEG signal was filtered in the bandwidth 0.16–3.5 Hz using finite-impulse-response (FIR) filters from the EEGLAB toolbox [69] (FIR band-pass filter, filter order corresponds to 3 cycles of the low frequency cut off). Next, positive to negative zero-crossings of the down-sampled signal were determined, and the consecutive crossings within an interval of 0.8–2 s (corresponding to 0.5–1.25 Hz) were selected. The negative and positive peaks within the aforementioned intervals were detected. The negative peak of each slow oscillation was determined if (1) its amplitude surpassed the subjects’ averaged negative peak by a factor of 1.25 and (2) the amplitude difference between the negative and positive peaks was 1.25 times larger than the averaged difference between the negative and positive peaks. These averaged values were determined for each channel (Fz and Cz) in N3 during the 150-min poststimulation interval. SO detection was the first step toward phase amplitude coupling (see 2.11).

### Time–frequency representations

From the time–frequency representations (TFRs) power fluctuations of individual slow and fast frequency bands were obtained for implementation in the phase amplitude coupling analyses (PAC, 2.11). The TFR algorithm was similar to the one previously employed by Ladenbauer et al. [54] In brief, per subject and electrode, TFRs were calculated from all detected artifact-free SO events within the range 5–20 Hz with a resolution of 0.25 Hz. A sliding window (in 10 ms steps; Hanning tapered) of variable length, dependent upon frequency was used (i.e. length constituted a full number of five cycles to ensure reliable power estimate (“mtmconvol” function of the FieldTrip toolbox) [70]. For each SO event, TFRs were normalized as difference to pre-event baseline (–2.5 to –1.2 s). TFR calculations were conducted ±3 s around SO trough to avoid edge effect in calculating of TFR.

### Phase amplitude coupling

An event-locked analyses as reported by Ladenbauer et al. [54] was employed to quantify the modulation of spindle amplitude with the phase of detected SOs (see 2.10). The time series of fluctuations in slow and fast spindle power around the SO trough (negative EEG half-wave) were obtained by averaging each bin of the TFR (see 2.10) across the respective frequency bands for each subject (see 2.8). To ensure proper phase estimation, both time series, the SO and the fluctuations in spindle power, were first filtered in the range of the modulating SO event (0.5–1.25 Hz; FIR band-pass filter, filter order of 3 cycles the low frequency cut off, EEGLAB toolbox). To avoid edge effects, we conducted zero-padding. Since the order of the filter is defined as 3 cycles of low cut off frequency (the lowest frequency is 0.5 Hz) and the minimum length of the signal should be 3 times the filter order: 3 × 3/0.5 = 18 s, we added 8 s zeros to each side of the ±1 s long signal. For each electrode separately, phase values were calculated for all time points around the SO trough and the corresponding slow and fast spindle power fluctuation using the Hilbert transform. The synchronization index (SI) was calculated between the two-phase value time series as:


$$\begin{array}{l} SI=\frac{1}{m}\sum_{j=1}^{m}e^{i\left[\theta_{SO}\left(j\right)-\theta_{SP}\left(j\right)\right]} \end{array}$$

where *m* is the number of time points (–1 s to +1 s around the SO trough), θ _SO_(_*j*_) is the phase value of the SO time series at time point *t*_*j*_, and θ _SP_(_*j*_) is the phase value of the fluctuations of the fast/slow spindle power time series at time point *t*_*j*_ [71]. Details of the procedure to compute the SI are shown in Figure S1.

**Figure 1.**
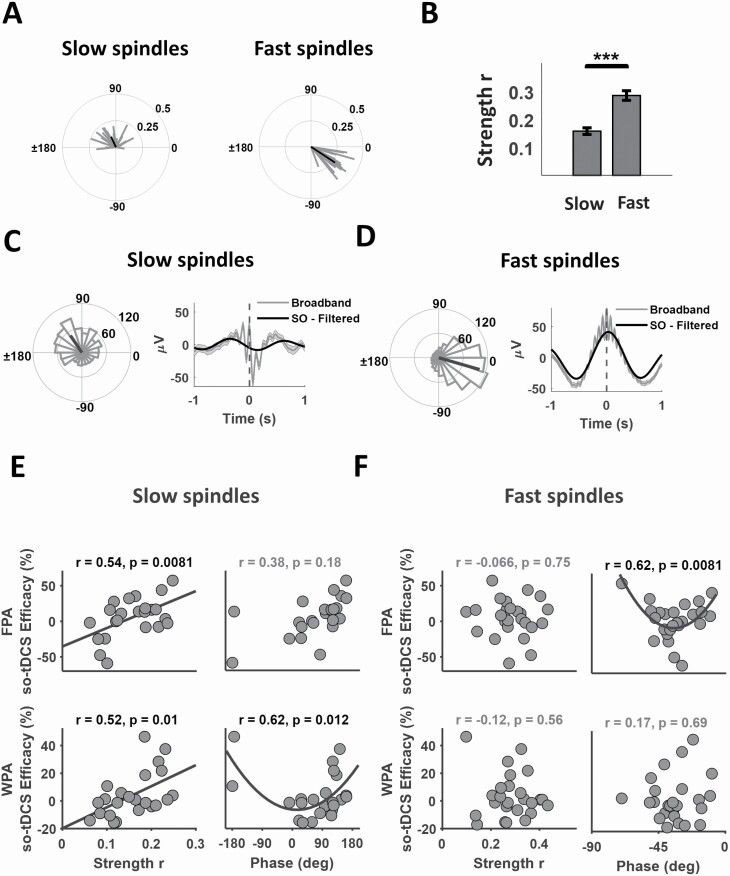
SO-spindle coupling measures predict stimulation efficacy on retention. (A) Circular histograms of average SI angles per subject (gray lines) and across subjects (black line) for SO-slow spindle coupling (left) and SO-fast spindle coupling (right). An angle of 0° indicates phase synchrony, and an angle of ±180° an anti-phase relationship. A value between −90 and 0° indicates that spindle power peaks before the SO peak. A value between ±180 and 90° indicates that spindle power peaks before the SO trough. At the individual subject level, the circular histograms of SI angles revealed significantly nonuniform distributions in 91% and 100% of cases, for SO-slow and SO-fast spindle coupling respectively (each *p* < 0.05, Rayleigh test). Average SI angles for SO-fast spindle coupling were more focused than for SO-slow spindle coupling (*p* < 0.00001, circular *k* test). (B) Average coupling strength of SO-fast spindle coupling was significantly larger than the coupling strength of SO-slow spindle coupling, *** *p* < 0.001, paired *t*-test. (C) Circular histogram of individual SI angles for SO-slow spindle coupling for one exemplary subject (*n* = 363 SO events, slow spindle frequency range: 9.78–11.78 Hz, Rayleigh test; *z* = 26.06, *p* < 0.00001). The thick line shows the direction of average SI angle of this subject across all SO events (left). For visualization, the plot on the right depicts the average of broadband EEG (0.16–33 Hz) time-locked to the maximum peak of detected slow spindles (*n* = 158) for the same subject (gray, cp. Methods, 2.12). Black line indicates the average signal filtered in the SO frequency band (0.5–1.25 Hz). Note, *t* = 0 corresponds to the maximum peak of detected slow spindles. (D) Same as (C), but for SO-fast spindle coupling (SOs, *n* = 352, fast spindles, *n* = 167, fast spindle frequency range: 12.67–14.67 Hz, Rayleigh test; *z* = 24.63, *p* < 0.00001). (E) so-tDCS efficacy (retention_STIM_ – retention_SHAM_) in the FPA and WPA tasks is correlated with SO-slow spindle coupling strength (left, *n* =23, Pearson correlation). Black solid line shows a linear fit to the data with significant correlation. Circular–linear correlation analyses between so-tDCS efficacy in the FPA and WPA tasks and slow spindle SI angle (right). To further visualize nonlinear circular-linear relationship for the significant correlations a quadratic fit is shown by black solid curve. (F) so-tDCS efficacy in the FPA task correlates with the fast spindle SI angle (*n* =25, circular–linear correlation). Note the SI angles represent the phase difference between the SO potential and spindle power. All EEG data were computed during baseline sleep. All *p*-values are uncorrected. Significant correlations after Benjamini–Hochberg correction for multiple comparisons are shown in black.

For each detected SO (see 2.9), resulting SI is a complex number of which the absolute value (*r*) indicates the strength of locking between the SO event and fluctuations in slow or fast spindle power. The corresponding SI angle represents the phase difference between SO potential and spindle power. For each subject (“individual” values), real and imaginary parts of the SI across all the detected SOs were averaged. In Figure 1, SI angles are shown for two exemplary subjects and averaged across subjects.

### Spindle detection

Spindle events were specifically detected to depict the agreement in phase between the employed method for PAC (see 2.11) and a method based on detection of spindle events. The spindle detection procedure was essentially identical to the one previously reported by Koo and colleagues [60]. After filtering the EEG signal within the individually determined spindle frequency bands (FIR band-pass filter, filter order of 3 cycles the low frequency cut off, EEGLAB toolbox), the root mean square (RMS) of the filtered signal was calculated using a moving window of 0.2 s, with a step size of 10 ms and subsequently smoothed using a moving window of 0.2 s. The spindle threshold value was set at 1.5 standard deviation (SD) of the filtered signal within the 150-min poststimulation period of the baseline sleep. Individual thresholds were determined separately for each channel, Fz and Cz. If the RMS signal remained above threshold for 0.5–3 s, the time frame was considered a spindle interval. The spindle event time (*t* = 0) is defined as the peak of the filtered signal.

### Slopes of the slow oscillation

SO slope was calculated from the EEG signal filtered in the frequency range of 0.16–3.5 Hz at Fz around each detected SO. SO slopes during up- to down-state (and down- to up-state) transitions were determined as the ratio between the absolute value of the SO trough, corresponding to the peak of the negative SO half-wave or down-state, and the time delay to the previous (next) zero crossing [39].

### Statistical analysis

Unless otherwise stated MATLAB (version 2017b) was used for all statistical analyses. Statistical analyses on the SI angles employed MATLAB CircStat toolbox [72]. The differences in the strength of SO-spindle coupling between slow and fast sleep spindles in the nonleaning, nonstimulation baseline sleep session were tested by paired *t*-tests. To verify circular nonuniformity of SI angles (both within and between subjects) we applied the basic Rayleigh test [73]. If the null hypothesis cannot be rejected, data reveal a circular uniform distribution. The concentration of SI angles of slow and fast spindles was compared with the parametric circular *k* test: a higher concentration of angles on circular data is translated into a larger resultant length of vector sum [74]. Equations are to be found in Fischer [75].

The relationship between strength of SO-spindle coupling and so-tDCS efficacy on retention, between the SO slope and so-tDCS efficacy, as well as between slow spindle peak frequency and strength of SO-spindle coupling were investigated via Pearson correlations. The data of two subjects with slow spindle power greater than the mean ± four times SD were excluded from all statistical analyses on SO-slow spindle coupling. All averaged values are reported as mean ± SEM.

To investigate whether the phase of SO-spindle coupling is related to so-tDCS efficacy and to find the correlation between slow spindle peak frequency and phase of SO-slow spindle coupling, a circular-linear (cl) correlation [76] was calculated according to the following equation (CircStat toolbox):


$$\begin{array}{l} p_{cl}=\sqrt{\frac{r_{xs}^{2}+r_{xc}^{2}-2r_{xs}r_{xc}r_{cs}}{1-r_{cs}^{2}}}, \end{array}$$

where *r*_*xs*_, *r*_*xc*_ and *r*_*cs*_ were defined as:

*r*_*xs*_ = corr(*x*, sin(alpha))

*r*_*xc*_ = corr(*x*, cos(alpha))

*r*_*cs*_= corr(sin(alpha), cos(alpha)).

In the above equations, *x* represents the linear variable (can be so-tDCS efficacy on retention, peak frequency or slope) and alpha represents the circular variable (SI angle).

The Benjamini–Hochberg procedure was used to control the false discovery rate (FDR) [77]. There were 12 tests for the correlation between SO-spindle coupling and so-tDCS efficacy on retention, and six tests for the correlation between SO slope and so-tDCS efficacy. Thus, we corrected for 18 multiple comparisons (Benjamini–Hochberg). For the control tests, i.e. correlations between SO-theta-/SO-slow spindle coupling measures and spindle peak /theta peak frequency/ task efficacy of Figure 3 and Table S7 multiple comparison corrections for in total 10 tests were made (Benjamini–Hochberg). The false discovery rate (FDR) was set at 0.05 unless mentioned otherwise. All reported *p*-values were uncorrected. Data available on request.

## Results

### Sleep parameters and behavior

Polysomnographic measures of the baseline night and in the two learning sessions, learning and retention performance were as reported previously (cp. Tables S1 and S2) [60]. Similarly, so-tDCs affected fast spindle power and density during the 150-min poststimulation period (Table S3).

### Stimulation efficacy on retention correlated with SO-spindle coupling measures

Conventional measures of sleep rhythms in baseline sleep are given in Tables 1, S1, S3. Figure 1, A reveals the typical phase and strength of coupling for the two spindle types: Average individual SI angles of slow spindles relative to SO were mostly in the upper half plane (0–180°), corresponding to the up-to-down transition, i.e. the negative-going EEG SO slope (mean SI angle across all subjects: 110.34 ± 10.21°). Unlike for slow spindle coupling, average individual-SI angles of SO-fast spindle coupling were all in the lower right quadrant (–90−0°), corresponding to the end of the down-to-up transition, i.e., before the SO peak (0°). The mean SI angle across all subjects here was equal to −33.54 ± 2.75°. As reported previously [42], average SI angles for SO-fast spindle coupling were more focused than for SO-slow spindle coupling (resultant vector length for SO-fast spindle coupling was 22.31, and for SO-slow spindle coupling 14.59; *F* =12.29, *p* < 0.00001, circular k test). SO-fast spindle coupling strength was significantly larger than that of SO-slow spindle coupling (fast spindle: *r* = 0.29 ± 0.017 versus slow spindle: *r* = 0.15 ± 0.011, *t*(22) = 8.01, *p* < 0.001, paired *t*-test; Figure 1, B).

**Table 1.** SO, slow and fast spindle properties

Sleep oscillations properties	Mean ± SEM
Slow spindle power (µV^2^)	0.70±0.90
Fast spindle power (µV^2^)	0.54±0.060
SO power (µV^2^)	172.14±19.20
Slow spindle density (per 30s)	1.48±0.068
Fast spindle density (per 30s)	2.20±0.092
SO density (per 30s)	3.52±0.10
Slow spindle duration (s)	0.76±0.010
Fast spindle duration (s)	0.76±0.010
SOs duration (s)	1.19±0.010
up-to-down-slope (μV/s)	809.82±101.51
down-to-up-slope (μV/s)	576.53±49.85

Mean (± SEM) of the conventional sleep rhythms during N3 of baseline sleep, i.e. within the interval of sleep corresponding to the 150-min poststimulation interval. Corresponding values for stimulation and Sham sessions are given in Table S3.

To investigate the ability of SO-spindle coupling measures of baseline sleep to predict stimulation efficacy we examined the correlation between so-tDCS efficacy on retention performance and endogenous SO-spindle coupling measures. SO-tDCs efficacy on retention of the FPA and WPA tasks correlated with SO-spindle coupling measures in baseline sleep. No correlation, however, was found for retention on the 2DL task (*p* > 0.1, see Table S4). For both FPA and WPA tasks so-tDCS efficacy correlated significantly with SO-slow spindle coupling strength (Pearson correlations, FPA: *r* = 0.54, *p* = 0.0081; WPA: *r* = 0.52, *p* = 0.010; Figure 1, E). For WPA, so-tDCS efficacy also correlated with SO-slow spindle coupling phase (circular-linear correlation, *r* = 0.62, *p* = 0.012; Figure 1, E), indicating that subjects with SI angles closer to 180° (SO trough) revealed greater so-tDCS efficacy. Although a similar distribution was found for the FPA task, the relationship was not significant (circular-linear correlation, *r* =0.38, *p* = 0.18; Figure 1, E).

Regarding SO-fast spindle coupling, for the FPA task only, a robust correlation of so-tDCS efficacy with SO-spindle coupling phase was measured (*r* = 0.62, *p* = 0.0081; Figure 1, F), indicating that the lowest so-tDCS efficacy was associated with SO-fast spindle coupling around −45°. SO-tDCS efficacy on WPA was not significantly correlated with SO-fast spindle coupling phase (circular-linear correlation, *r* = 0.17, *p* = 0.69; Figure 1, F). Importantly, SO-tDCS efficacy did not correlate with any conventional SO or fast/slow spindle properties such as power, density or event duration, after correction for multiple comparisons (Table 2).

**Table 2.** SO-tDCS efficacy and conventional EEG measures

		WPA		FPA		2D-L	
		*r*	*p*-value	*r*	*p*-value	*r*	*p*-value
	Power	0.14	0.52	0.32	0.12	0.10	0.62
SO	Density	−0.10	0.62	0.18	0.40	−0.15	0.47
	Duration	0.20	0.34	−0.13	0.55	−0.16	0.44
	Power	0.13	0.55	0.15	0.50	0.14	0.54
Slow spindle	Density	0.0084	0.97	−0.05	0.82	0.041	0.85
	Duration	−0.34	0.11	−0.18	0.42	0.44	0.036
	Power	−0.14	0.52	0.20	0.34	0.25	0.22
Fast spindle	Density	−0.025	0.90	−0.077	0.71	0.031	0.88
	Duration	−0.11	0.60	−0.13	0.55	0.39	0.056

Pearson correlation coefficients r and uncorrected *p*-values for correlations between so-tDCS efficacy and conventional measures of slow oscillations (SO), slow and fast spindles during N3 of baseline sleep for the three declarative memory tasks. WPA, word paired-associate; FPA, figural paired-associate, 2-DL, 2D-object location.

Together, for so-tDCS efficacy on retention, strong evidence is found toward a positive correlation with strength of SO-slow spindle coupling during N3 of in baseline sleep. Interestingly, SO-fast spindle coupling only correlated with so-tDCS efficacy on retention of the FPA task.

### Relationship between stimulation efficacy on retention and SO slope

**Figure 2.**
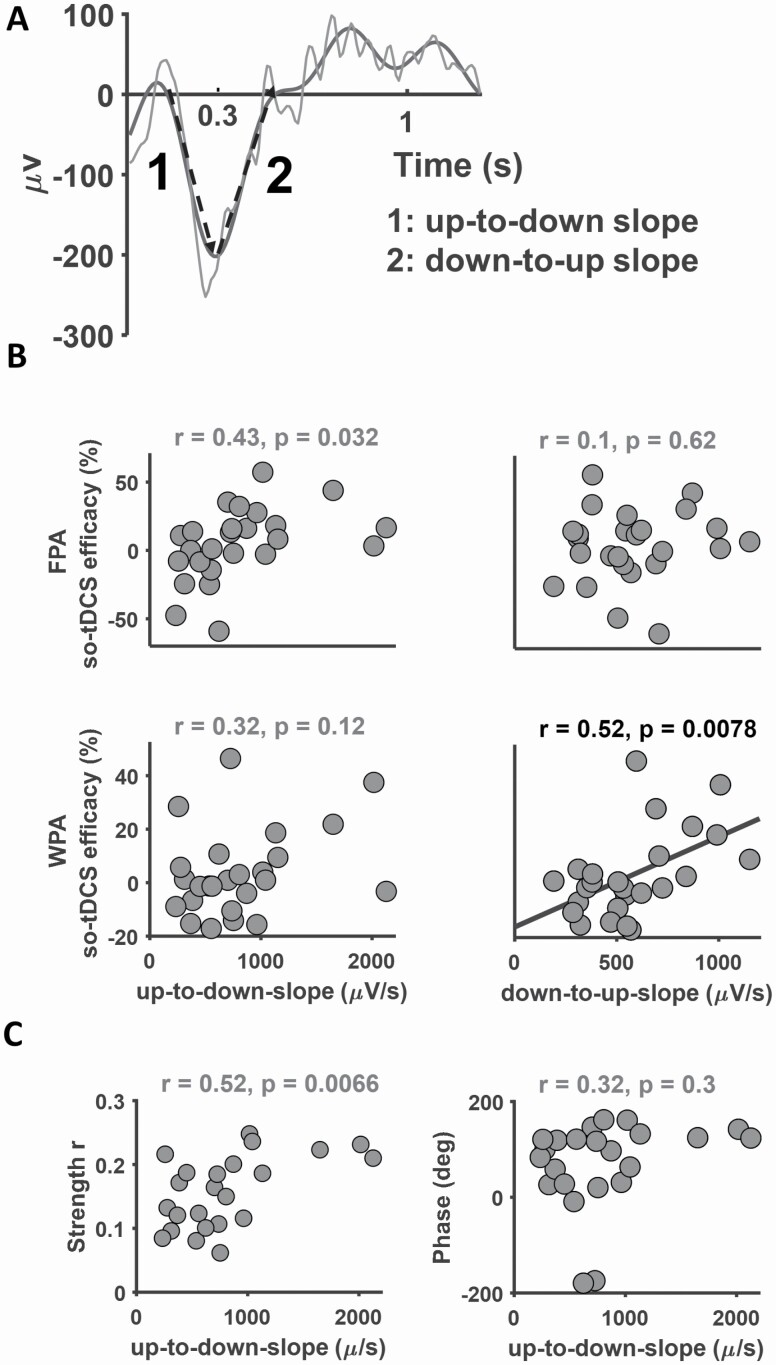
SO slope predicts stimulation efficacy on retention. (A) SO up-to-down and down-to-up slopes are defined on an exemplary SO (gray curve). SO slopes (dashed lines) were obtained from the EEG signal filtered in the 0.16–3.5 Hz frequency range (thin black curve, cp. Methods 2.13). (B) Relationship between so-tDCS efficacy on retention in the FPA and WPA tasks and SO up-to-down slope (left) or SO down-to-up slope (right). Black solid line shows a linear fit to the data with a significant correlation. Pearson correlation coefficients and *p*-values are displayed on each correlation plot. (C) Relationship between SO-slow spindle coupling strength (left, Pearson correlation) and phase (right, circular-linear correlation) and SO up-to-down slope. Note the SI angles represent the phase difference between the SO potential and spindle power. All EEG data were computed during baseline sleep. All *p*-values are uncorrected. Significant correlations after Benjamini–Hochberg correction for multiple comparisons are shown in black.

To investigate the potential ability of SO slopes of baseline sleep to predict stimulation efficacy we examined their correlation. Results aim to lend information on the dependence of stimulation efficacy on endogenous network activity at the transition between SO states. A sketch of SO slopes in relation to SO up and down states is given in Figure 2, A. SO-tDCS efficacy on retention in the WPA task revealed a significant positive correlation with SO down-to-up slope (*r* = 0.52, *p* = 0.0078, Pearson correlation, FDR set to 5%; Figure 2, B). SO-tDCS efficacy on retention in the FPA task did not correlate with the SO down-to-up slope, rather, a closer relationship to the opposite, SO up-to-down, slope may exist. This correlation only withstood multiple comparison testing with FDR set at 10% (*r* = 0.43, *p* = 0.032; Figure 2, B). No significant correlation with SO slope was found for so-tDCS efficacy on retention in the 2DL task (*p* > 0.1; see Table S4 for test results of the 2DL task).

Since especially slow spindles mostly occur during the falling phase of the down state, we further investigated the correlation between SO-spindle coupling measures and SO slope. There appeared to be a positive relationship between SO-slow spindle coupling strength and SO up-to-down slope (*r* = 0.52, *p* = 0.0066, Figure 2, C), which however only reached significance when the FDR was set at 10%. There was no significant correlation between SO-slow spindle coupling phase and SO up-to-down slope, nor between either SO-slow spindle coupling measure and SO down-to-up slope, or between either SO-fast spindle coupling measure and SO slope (*p* > 0.05). These results suggest that SO slope and SO-spindle coupling measures are independent.

Together, so-tDCS efficacy on retention in the WPA task reveals a strong relationship to spontaneous SO down-to-up slope in baseline sleep.

### SO-slow spindle coupling and slow spindle peak frequency

Above we reported that so-tDCS efficacy on the WPA and FPA tasks correlated with features of SO-slow spindle coupling. Recently, a debate on the distinction between EEG slow spindles and intracranial theta bursts [78], two rhythms, both occurring during the up-to-down SO slope has emerged. Thus, we investigated for any dependence of SO-slow spindle coupling measures on peak frequencies within the selected slow spindle band. Furthermore, we detected peak frequencies within the theta (5–8 Hz) frequency band of NREM sleep (preferably within N3; Table S6) and analyzed SO coupling to explore similarities and differences.

**Figure 3.**
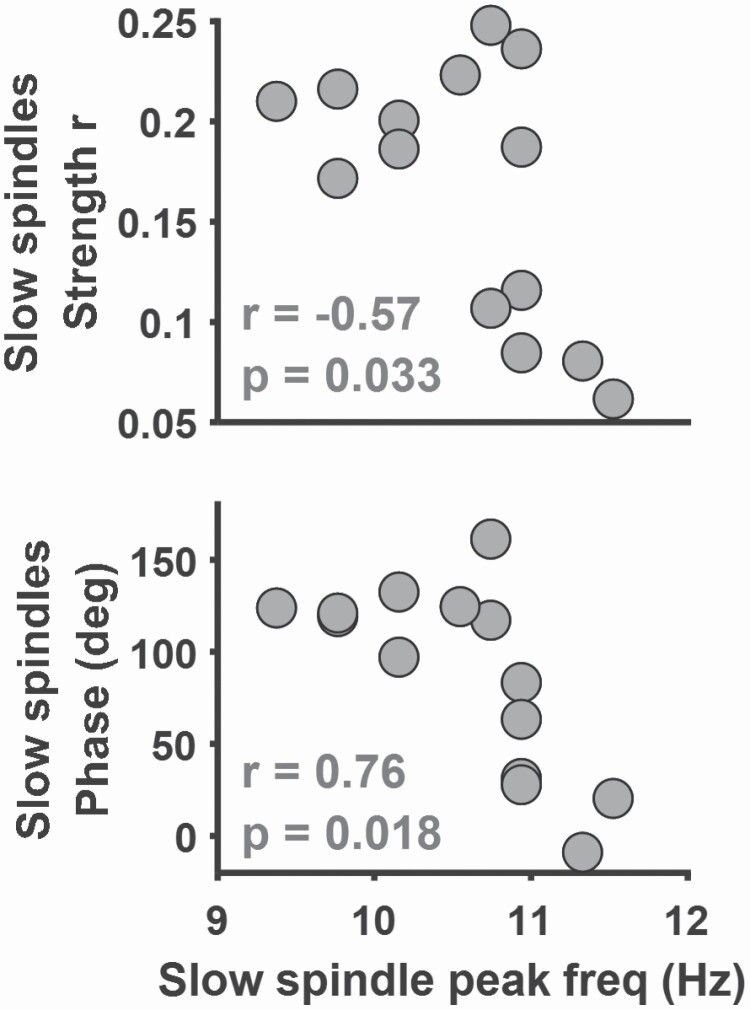
Relationship of SO-slow spindle coupling to slow spindle peak frequency. Pearson correlation between SO-slow spindle coupling phase (top) and SO-slow spindle coupling strength (bottom) and slow spindle peak frequency, *n* = 14. Note the SI angles represent the phase difference between the SO potential and spindle power. All EEG data were computed during baseline sleep. All *p*-values are uncorrected. Correlations in gray indicate the threshold for significance was not met after FDR correction.

Firstly, as revealed in Figure 3, all individual slow spindle peak frequencies were indeed above 9 Hz, i.e. either above or at the upper limit of the theta frequency band. Secondly, the plots show that lower slow spindle peak frequency coincided with increased SO-slow spindle coupling strength (*r* = −0.57, *p* = 0.033, Pearson correlation), and with SO coupling phases closer to SO trough (180°; *r* = 0.76, *p* = 0.018, circular-linear correlation), but significance was not maintained at an FDR of 5%. Thirdly, unlike SO-slow spindle coupling, SO-theta coupling was correlated neither with so-tDCS efficacy on any of the tasks nor with theta peak frequency, for those subjects revealing clear theta peaks (Tables S5, S6).

## Discussion

In sleep, spontaneous EEG SO-spindle coupling gives information on thalamo-cortical information transfer, which may contribute, to neuroplastic mechanisms of memory consolidation. Our investigations revealed that within a nonlearning baseline sleep session SO-spindle coupling and SO slope, a potential indicator of synaptic strength, were of predictive value for the efficacy of anodal so-tDCS stimulation on memory consolidation of word and figural paired-associate tasks. Predictive quality of baseline sleep EEG properties has been researched in several contexts [8, 79], e.g. on next morning cognitive abilities [45], on cognitive abilities in response to sleep deprivation [80, 81], or of spindle features on cognitive abilities [18, 82]. Increased SO-spindle coupling strength from childhood to adolescence was correlated positively with overnight memory consolidation [65]. However, we are not aware of any study revealing that the efficacy of weak electric stimulation (tACS, o-tDCS, tDCS) or other exogenous, e.g. sensory stimulation, during sleep depends on baseline EEG parameters. Our findings indicate that baseline network features can relate to facilitated neuroplasticity expressed in improved retention performance.

Intraindividual differences in, and predictive quality of baseline sleep may reflect intraindividual moderately persistent cortical states rooted in neocortical microstructure [79, 83–85], functional connectivity [86], glymphatic or other homeostatic function. Our finding that SO-slow spindle coupling strength was of predictive value for so-tDCS efficacy on retention of both paired-associate tasks, supports the concept that behaviorally relevant neuroplastic changes are dependent upon a temporally localized interplay within thalamo-cortical and putatively hippocampal circuits. This interplay, on the other hand, operates within the framework of intraindividual or rather stable cortical states.

Regarding the phase to which spindles couple to SO, for slow spindles so-tDCS efficacy on WPA-retention was increased on occurrence of spindle coupling closer to SO trough (−180°) during baseline sleep. For fast spindles the circular correlation suggests increased so-tDCS efficacy on FPA-retention for coupling closer to SO peak (at 0°) than to 45°. Indeed, we had expected that subjects revealing fast spindle coupling closer to SO peak would reveal increased so-tDCS efficacy on retention, since enhanced memory consolidation in young vs. older subjects coincided with SO-fast spindle coupling closer to SO peak, presumably reflecting more efficient neural communication [27]. We cannot decisively explain the absence of any significant SO-fast spindle phase coupling for WPA, although it is to remark here that the original data in Koo et al. [60] did not find an overall improvement on the WPA task after so-tDCS.

SO-tDCS efficacy on WPA-retention also revealed a positive correlation to the SO down-to-up slope, i.e. with the onset of neuronal activity leading to the up state. During this SO transition phase, rate of SPWRs and locus coeruleus firing are typically increased [87, 88], possibly indicating that such activity during baseline sleep has a potential benefit on ability toward neuroplastic responsiveness, but see Todorova [89]. Of interest, is that the so-tDCs efficacy on FPA-retention revealed a closer association with the SO slope of the up-to-down state transition. These latter results we take as an indication that so-tDCS-efficacy on retention of the two tasks could be biased, i.e. more strongly related to activity occurring during the SO falling versus rising phase. It would be too speculative to make any assumption on specific neocortical and/or neocortical-subcortical network interactions. Moreover, activity of the up-to-down and down-to-up states are not independent, resulting from local activity and long-range neuronal connections as well as from nonneuronal responses [35, 90–94]. We suggest that future studies investigate whether information on the interplay of sequential neural circuitry during the falling and rising phase of the SO can be related to so-tDCs efficacy, and that studies confirm ties between baseline sleep and task-dependent so-tDCS efficacy. A limitation in the present data is that Koo et al. found a correlation of so-tDCS efficacy on FPA-retention with increased memory quotient [95], and performance on the FPA task after so-tDCS was enhanced only for subjects with increased memory quotient indicating that the effect of so-tDCS was low [60]. The interaction of memory quotient with characteristics of baseline sleep is not introduced in this study.

The steep SO slopes may trivially result in the occurrence of nonzero activity at higher frequencies resulting in oscillations at theta and slow spindle frequency range [78, 96], thus potentially producing an artefactual SO-spindle coupling measures. In our analyses, SO-slow spindle coupling was distinct from and not mirrored by SO-theta coupling; our detected slow spindle peak frequencies were distinct from theta peak frequencies in power spectra, and SO-theta measures did not correlate with so-tDCS efficacy. Thus, although SO-theta coupling in some subjects was discerned in our scalp recordings as found by Gonazalez et al. [78], coupling behavior was not predictive for so-tDCS efficacy. Klinzing et al. [24] similarly concluded that their observed changes in slow spindle and theta activity coupled to sleep slow oscillations were independent [24]. One major differences between the latter study and ours as compared to Gonzalez at al. is that we analyzed deep N3 sleep, whereas theta-bursts were found most prominent during N2 sleep [78]. Levels of endogenous neurochemicals, phasic activity of brainstem loci, and changes across time in homeostatic process are likely sources of sub- or micro-NREM sleep states [17, 97–99]. Our finding that both SO-slow spindle coupling phase and strength change systematically dependent upon slow spindle peak frequency, make closer examination of microcircuits during the SO up-to down slope specifically intriguing [21, 35, 89, 90]. Thus, we believe more intense research employing cortical far-field, local-field, unit recordings and simultaneous subcortical recordings is required to resolve neural processes and functional relevance of rhythms during the SO up-to-down slope.

As mentioned above a limitation of this study is that overall efficacy of so-tDCS was weak. A systematic topographical analysis was not undertaken. For instance, the lack of any predictive measure of baseline sleep for tDCS efficacy on the 2D-object location task may be attributed to the spatial nature of this task, for which so-tDCS (targeting the frontocortical region) was not effective. We based our hypothesis on results of a previous paper [60], and therefore used these data. It would be too simplistic from our findings to generalize on differences between figural- and word-pair learning and memory tasks. Further studies on the precise encoding and consolidation processes of these tasks are required.

Conclusions from our results are not only scientifically, but also of potential clinical relevance. For one, SO-spindle coupling parameters and steepness in SO slope of baseline sleep may prove to be relevant markers for the susceptibility of an individual to anodal so-tDCS, and/or modulation of the consolidation processes. Confirmation of such relationships would contribute to precision medicine research. Secondly, so-tDCs efficacy on retention performance of the word and figural paired-associate tasks differed in part regarding their correlation with SO-spindle subtypes. Thus, underscoring recent investigations stating a need for future studies to look more specifically into the relationship between the nature of stored information and the specific constellation of brain electric activity and pathways involved [5, 100, 101]. Together, our electrophysiological and neurocognitive findings as well as those of others [25, 42, 102] suggest that the diverse, possibly complimentary processes within NREM sleep have predictive quality for efficacy of weak electric current stimulation or predominance of strategy used in the processing learned material. Future investigations must increasingly take interindividual differences into account.

## Supplementary Material

zsab127_suppl_Supplementary_Materials
