# Supplementary material for: Spontaneous slow oscillation—slow spindle features predict induced overnight memory retention
Source: Sleep. 2021 May 18;44(10):zsab127. doi: 10.1093/sleep/zsab127 (PMC8503833; doi:10.1093/sleep/zsab127)
Supplement: zsab127_suppl_Supplementary_Materials [file zsab127_suppl_supplementary_materials.pdf]

Spontaneous slow oscillation - slow spindle features predict induced overnight memory retention

Fereshteh Dehnavi[a], Ping Chai Koo-Poeggel[b,c], Maryam Ghorbani[a,d,*], Lisa Marshall[b,c,*]

[a] Department of Electrical Engineering, Ferdowsi University of Mashhad, Mashhad, Iran, Postal code: 9177948974

[b] Institute of Experimental and Clinical Pharmacology and Toxicology, University of Lübeck, Ratzeburger Allee 160, Bldg 66, 23562 Lübeck, Germany

[c] Center for Brain, Behavior and Metabolism, University of Lübeck, 23562 Lübeck

[d] Rayan Center for Neuroscience and Behavior, Ferdowsi University of Mashhad, Mashhad, Iran, Postal code: 9177948974

* Corresponding authors:

Lisa Marshall
University of Lübeck
Institute of Experimental and Clinical Pharmacology and Toxicology
Center for Brain, Behavior and Metabolism
Ratzeburger Allee 160, Bldg 66
23562 Lübeck, Germany
E-mail: lisa.marshall@uni-luebeck.de

or

Maryam Ghorbani
Department of Electrical Engineering,
Ferdowsi University of Mashhad,
Mashhad, 9177948974
Iran
E-mail: maryamgh@um.ac.ir

Supplementary material

SO-slow spindles coupling

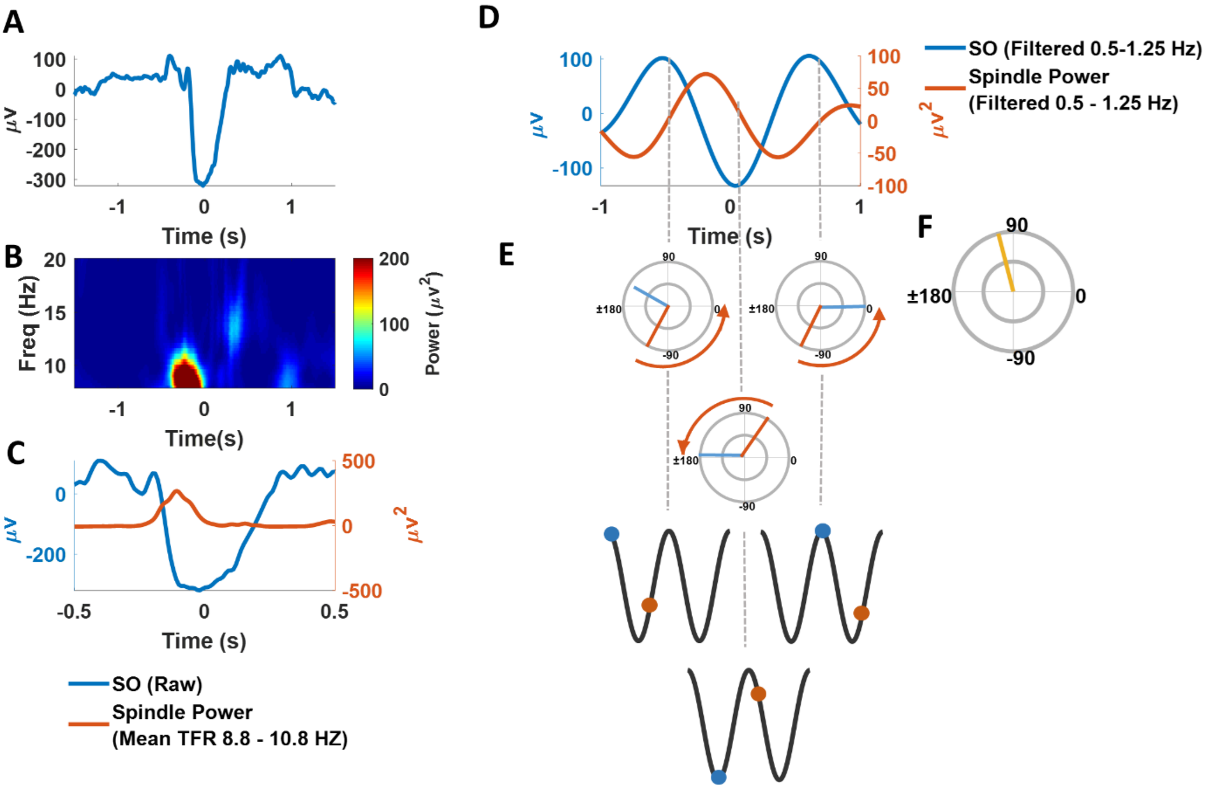

SO-fast spindles coupling

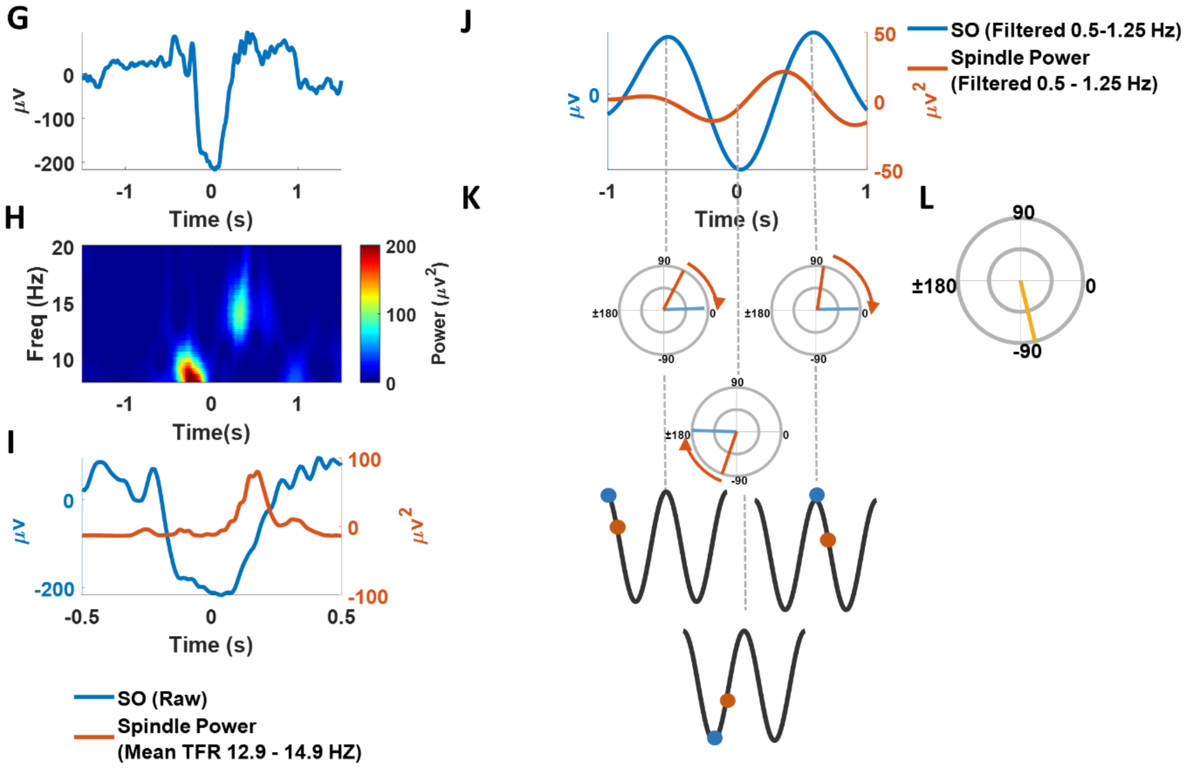

Figure S1. Procedures to compute the SO-spindle coupling measures. A) Broadband EEG time-locked to one exemplary SO event. B) TFR around the SO event was calculated and normalized as the difference to pre-event baseline (-2.5 to -1.2 s). C) Slow spindle power (red curve) was obtained by averaging each bin of the TFR across the respective frequency bands. D) To obtain the phase difference between time courses of SO potential and spindle power, first 8 s zeros to each side of the ± 1 s long signal were added to avoid edge effects. Then, SO potential and spindle power were filtered in 0.5-1.25 Hz. The phase of both signals was obtained using the Hilbert transform. For visualization, the phases of the two signals at three time points are shown. For the complete analyses, the phase differences between the SO potential and spindle power are obtained at each bin (0.1 s). F) The synchronization index (SI) was calculated as the circular mean of the phase differences in (E) across all time points (± 1 s around the SO event). For each detected SO, resulting SI is a complex number of which the absolute value indicates the strength of locking between the SO event and slow spindle power. The corresponding SI angle represents the phase difference between SO potential and spindle power. (G)-(L) similar to (A)-(F) but for fast spindles. Fz was used for SO potential, Fz and Cz channels were used for slow and fast spindles, respectively.

Table S1. Sleep parameters during the three experimental conditions.

Parameters	CONT	SHAM	STIM
		Nocturnal sleep	
Total recording time	437.6 ± 3.0	437.2 ± 3.9	443.3 ± 3.1
Total sleep time	386.6 ± 5.0	378.2 ± 4.8	383.7 ± 3.9
Total wake time	24.4 ± 1.6	28.3 ± 2.0	28.6 ± 2.0
Sleep efficiency	88.3 ± 0.8	86.5 ± 0.9	86.6 ± 0.9
% N1	4.0 ± 0.5	3.7 ± 0.3	4.4 ± 0.5
% N2	44.7 ± 2.2	43.1 ± 2.1	44.0 ± 2.1
% N3	30.0 ± 2.4	32.9 ± 2.6	31.4 ± 2.5
% REM	21.2 ± 0.9	20.4 ± 1.2	20.1 ± 1.0
% NREM	74.7 ± 0.9	76.0 ± 1.3	5.4 ± 1.1
Sleep latency	11.5 ± 1.1	18.7 ± 2.1*	19.6 ± 2.3*
REM latency	111.7 ± 13.0	118.2 ± 8.6	116.1 ± 9.5
		150 min post-stimulation period	
N2 [min]	49.16 ± 4.16	48.26 ± 3.54	51.46 ± 4.61
N3 [min]	65.52 ± 4.95	68.14 ± 4.44	66.56 ± 5.04

Sleep latency is defined as the time from lights-off to the first occurrence of sleep stage 1 followed by sleep stage 2. Subjects revealed significantly longer sleep latency in both STIM and SHAM as compared to CONT. There were no significant differences in any other sleep parameters between the three conditions. * $p < 0.05$, for comparisons between conditions (N = 25). Values are given in mean ± SEM.

Table S2: Learning performance and retention.

Task	Learning (Number)		Retention (%)	
	SHAM	STIM	SHAM	STIM
WPA	43.48 ± 2.00	42.24 ± 2.15	27.51 ± 3.26	31.08 ± 3.82
FPA	11.36 ± 0.48	11.48 ± 0.48	0.84 ± 3.78	5.78 ± 3.80
2-DL	10.72 ± 0.30	11.56 ± 0.36	-5.21 ± 3.64	-6.02 ± 3.96

WPA, word paired-associate; FPA, figural paired-associate, 2-DL, 2D-object location (n=25)

Table S3. SO, slow and fast spindle properties during N3 in 150 min post-stimulation.

Sleep Oscillations Properties	SHAM	STIM	p-value
**Slow Spindle Power (μV²)**	0.68 ± 0.8	0.70 ± 0.8	0.68
**Fast Spindle Power (μV²)**	0.52 ± 0.052	0.58 ± 0.062	0.013*
**SO Power (μV²)**	172.10 ± 18.48	165.39 ± 19.61	0.42
**Slow Spindle Density (per 30s)**	1.41 ± 0.10	1.57 ± 0.15	0.29
**Fast Spindle Density (per 30s)**	2.13 ± 0.12	2.38 ± 0.11	0.047*
**SO Density (per 30s)**	3.57 ± 0.18	3.45 ± 0.18	0.52
**Slow Spindle Duration (s)**	0.76 ± 0.011	0.77 ± 0.011	0.57
**Fast Spindle Duration (s)**	0.76 ± 0.0098	0.77 ± 0.011	0.35
**SOs Duration (s)**	1.20 ± 0.0097	1.20 ± 0.013	0.54
**up-to-down-slope (μV/s)**	621.92 ± 76.62	821.43 ± 62.12	0.019*
**down-to-up-slope (μV/s)**	613.08 ± 71.71	662.52 ± 63.06	0.61

Mean ± SEM. *$p < 0.05$, paired t-test.

Table S4. Relationship of so-tDCS efficacy on the 2D-object location task with SO-spindle coupling measures and SO slope

Measure		Correlation Coefficient r	p-value
SO-slow	Strength	-0.042	0.85
	Phase	0.29	0.38
SO-fast	Strength	-0.11	0.62
	Phase	0.40	0.13
Slope	down-to-up	-0.17	0.40
	up-to-down	-0.09764	0.64

For strength and slope Pearson correlations were used; for phase non-linear circular correlations (n=25)

Table S5. The detected peak frequency of slow, fast spindles and theta activity for all subjects.

Subject	Slow Spindle Peak	Fast Spindle Peak	Theta Peak
1	*	13.9	6.6
2	*	12.7	6.3
3	9.8	13.9	5.7
4	10.2	12.9	6.9
5	**	12.7	6.7
6	10.9	12.9	6.5
7	11.3	13.5	6.5
8	***	13.3	*
9	*	15.0	7.7
10	**	13.9	8.0
11	11.5	13.7	5.7
12	**	14.1	7.8
13	10.9	13.5	*
14	10.7	13.9	6.8
15	9.8	13.7	7.8
16	10.7	13.7	7.8
17	10.2	13.9	6.8
18	10.9	13.5	6.8
19	*	14.6	6.6
20	**	13.5	5.7
21	10.9	13.1	6.84
22	10.6	13.9	6.8
23	**	13.7	7.4
24	***	14.1	7.8
25	9.4	13.7	7.6
Mean±SEM	10.6±0.16	13.6±0.11	6.9±0.15

* No detectable peak in the power spectra, ** slow spindle peak was larger than 11.5 Hz, *** Subject was excluded

Table S6. Relationship between so-tDCS efficacy and SO-theta coupling

SO-theta measures	so-tDCS efficacy on WPA		so-tDCS efficacy on FPA		so-tDCS efficacy on 2DL		Theta peak frequency	
	r	p-value	r	p-value	r	p-value	r	p-value
Strength	-0.025	0.90	0.11	0.61	-0.18	0.38	0.073	0.94
Phase	0.16	0.72	0.33	0.27	0.43	0.10	-0.066	0.77

Correlation coefficients r and p-values for correlations between so-tDCS efficacy with SO-theta coupling measures for the three declarative memory tasks. WPA, word paired-associate; FPA, figural paired-associate, 2-DL, 2D-object location (n=25 for the correlation with so-tDCS efficacy, n=23 for the correlation with theta peak frequency).
